# Air Quality and Climate Change: A Delicate Balance

**DOI:** 10.1289/ehp.123-A148

**Published:** 2015-06-01

**Authors:** John H. Tibbetts

**Affiliations:** John H. Tibbetts, based in Charleston, SC, is former editor of *Coastal Heritage*, the magazine of the South Carolina Sea Grant Consortium.

As researchers consider the potential health impacts of a warming planet, the relationships between climate change and air pollutants become increasingly important to understand. These relationships are complex and highly variable, depending on local conditions.

Dust, allergens, soot, water vapor, and other particles and gases in the atmosphere are constantly interacting and forming new mixtures, often with the influence of heat and ultraviolet radiation. Many direct human health effects of these airborne agents have been well characterized. Some of these agents also have greenhouse properties, contributing to the overall warming of the planet, while others impart cooling effects. Climate change and air pollution are thus inextricably intertwined.

**Figure:**
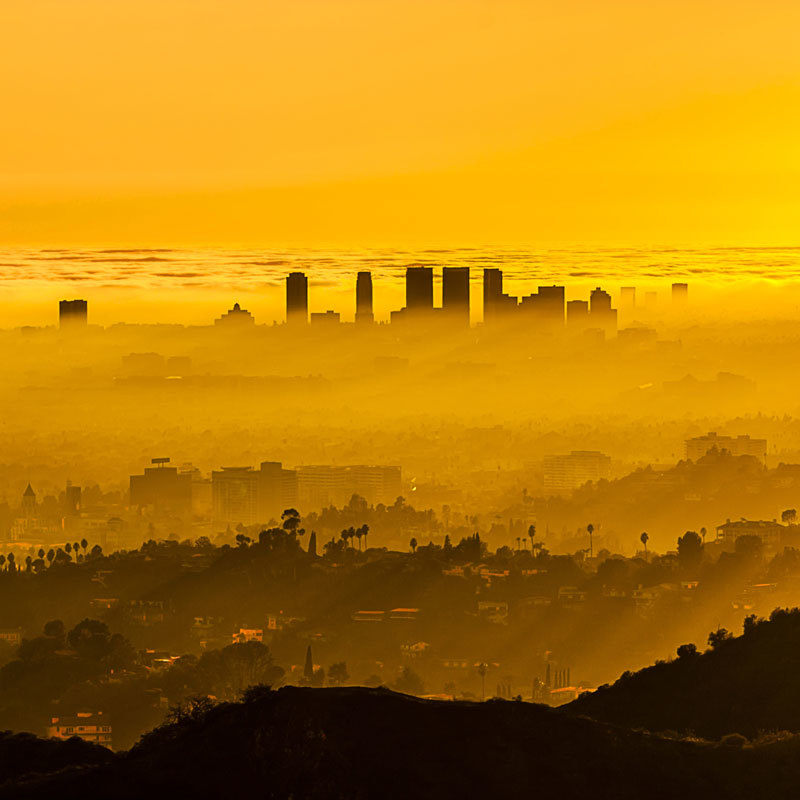
Climate change and air pollution are inextricably intertwined, so fighting one often produces gains against the other. © Carl Larson Photography/Getty Images

In the run-up to the United Nations Climate Change Conference to be held in Paris this December, stakeholders in the public health and government arenas are hammering out strategies to reduce emissions of short-lived climate pollutants.^1^ Many of these strategies will have the added benefit of improving health outcomes related to ambient air pollution.

## Ozone: The Good, the Bad, and the Ugly

Ground-level ozone (O_3_) is one of the major air pollutants discussed in terms of climate change. Some refer to O_3_ in terms of “the good, the bad, and the ugly,” says Megan L. Melamed, executive officer of the International Global Atmospheric Chemistry Project. The ozone layer located in the stratosphere, which protects human life from harmful ultraviolet radiation, is the “good.” Ground-level O_3_, with its myriad adverse health effects, is the “bad.” O_3_ also acts a short-lived climate pollutant, contributing to the greenhouse effect—the “ugly.”

Although higher temperatures are associated with elevated ground-level O_3_,^2^ ozone events require sunlight. Overall, the Intergovernmental Panel on Climate Change predicts, warmer temperatures and increased water vapor abundance will reduce baseline concentrations of ground-level O_3_—a positive development, given the health harm this pollutant can do.^3^ But other factors are likely to intensify O_3_ production in polluted areas, especially during heat waves and drought.^3^

O_3_ is created by chemical reactions between ultraviolet radiation and precursor air pollutants, including oxides of nitrogen (NO_x_), carbon monoxide, and volatile organic compounds (VOCs). These precursors come from anthropogenic sources such as automobile emissions, gasoline vapors, and power plants. They also come from natural sources, including vegetation (in the case of VOCs) and lightning (in the case of NO_x_).^4^

The smell of O_3_ is familiar to many city dwellers during the dog days of summer. The summer air seems to thicken when a high-pressure weather system stalls over a city, baking streets in blazing temperatures under cloudless skies. Winds die down, and hot city air is trapped under a dome of high pressure (or “heat dome”), concentrating pollutants near the ground in what’s called a stagnation event. These conditions are ideal for producing ground-level O_3_.

Ground-level O_3_ can be formed anywhere in the troposphere, the lowest layer of the atmosphere, which ranges from the surface up to elevations around 10–15 km, depending on latitude and season. O_3_ is also formed in the lower stratosphere, the next higher level of the atmosphere, up to elevations around 50 km.

Cities can have lower levels of O_3_ than one might expect, based on the amount of precursors produced by urban sources. Interaction with traffic-related NO_x_ emissions transforms a portion of urban O_3_ to O_2_, the most stable form of oxygen. Furthermore, once a stagnation event ends and winds resume, large concentrations of urban O_3_ and precursor pollutants can be transported into surrounding rural areas hundreds of miles downwind. O_3_ concentrations therefore tend to peak downwind of major cities, not in them.^5^

**Climate Change and Air Pollution: Interconnected Health Effects**

**Figure:**
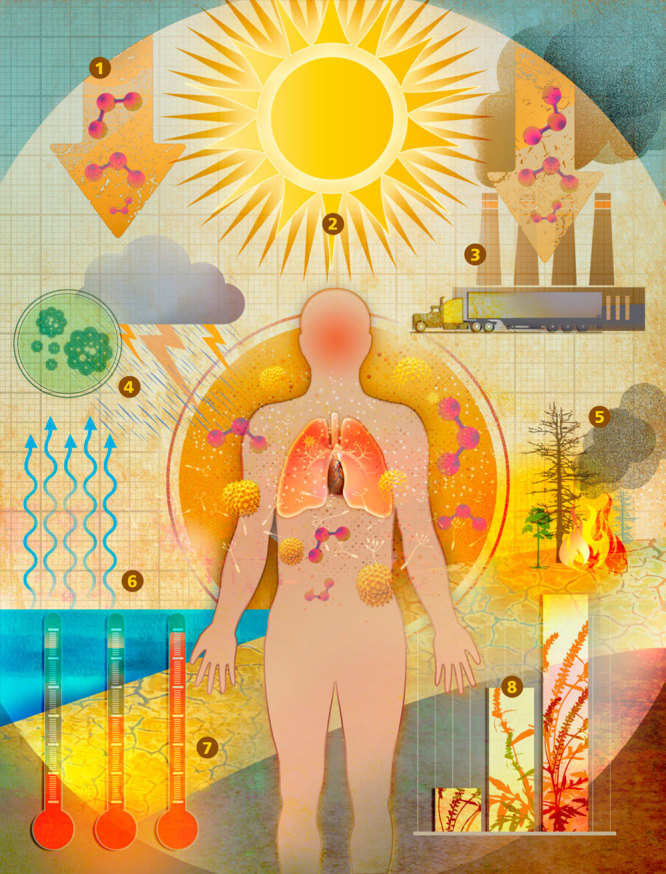
© Roy Scott

1. **ATMOSPHERIC PRESSURE**

When a dome of high atmospheric pressure stalls over an area, it concentrates pollutants close to the ground. High pressure also has been associated with a greater occurrence of cardiac arrhythmia^28^ and myocardial infarction,^29^ independent of other risk factors.

2. **HEAT AND SUNLIGHT**

Higher temperatures and ultraviolet radiation interact with precursor pollutants to produce ground-level O_3_. Exposure to O_3_ can cause shortness of breath, wheezing, coughing, lower respiratory tract infection, decreased lung function, airway injury and inflammation, and premature death.^30^^,^^31^ Even small increases in O_3_ beyond background levels may harm human health.^32^ Short-term hikes in O_3_ concentrations over 1–2 days have been associated with an increase in acute coronary events in middle-aged adults without previously diagnosed heart disease independently of meteorological elements.^33^

Heat stress and higher temperatures can also contribute to greater cardiovascular morbidity and mortality,^34^ probably via autonomic changes, dehydration, endothelial cell damage, and increased blood viscosity and platelet and red blood counts.^35^

3. **ANTHROPOGENIC EMISSIONS**

Emissions such as nitrogen oxides and volatile organic compounds contribute to O_3_ formation. In addition to causing direct human health effects, many emitted pollutants act as greenhouse gases, while some others, such as sulfur dioxide, have cooling properties.

4. **INCREASED PRECIPITATION**

Warming temperatures mean air is capable of holding more moisture. Increased atmospheric vapor combined with low pressure can result in more severe storms.^25^ Rain is necessary to clear pollutants from the air, but too much rain coming down too fast increases the risk of flooding. When homes are flooded, they are more susceptible to mold outbreaks. Exposure to mold toxins can cause respiratory illnesses.^36^

5. **WILDFIRES**

Hotter temperatures and drought contribute to increased risk of wildfires. Wildfire smoke contains more than 10,000 substances, which can travel long distances and affect large populations for days to months.^37^ Air pollution from wildfires drives up the numbers of hospitalizations and emergency department visits^38^ and causes an estimated 339,000 premature deaths per year worldwide.^39^

6. **INCREASED HUMIDITY**

Hotter, more humid weather tends to irritate airways, making breathing more difficult for many people with asthma and other respiratory ailments.^40^^,^^41^^,^^42^ Spikes in temperature and humidity are associated with increased emergency department visits for asthma attacks, especially in children.^42^ Such circumstances also are associated with ventricular arrhythmia^28^ and myocardial infarction.^35^

7. **DROUGHT**

Increased evaporation can be a major factor in heat waves and associated forest fires and droughts.^43^ WIthout rain, air becomes choked with dust, smoke, and other pollutants that can exacerbate or cause asthma, rhinosinusitis, chronic obstructive pulmonary disease, and lower respiratory tract infections.^10^^,^^11^

8. **POLLEN**

Airborne pollen, mold spores, and dust can trigger respiratory illnesses such as asthma, allergic rhinitis, conjunctivitis, and dermatitis.^14^ High pollen counts have been linked in several studies to increases in asthma emergency-department visits.^44^ Children are particularly susceptible to most allergic diseases.^45^

## Hotter, Drier Conditions

Hotter, drier conditions are by no means the only change expected with global warming. However, they do play significant roles in terms of degraded air quality.

Higher surface temperatures combined with natural stagnation events can intensify evaporation from soils and evapotranspiration from plants. An increase in carbon dioxide (CO_2_) and other greenhouse gases in the atmosphere provides just a little extra heat to a stagnation event, says Kevin E. Trenberth, a senior scientist with the National Center for Atmospheric Research in Boulder, Colorado. “If you accumulate this extra heat over a week or month,” he says, “it dries things out more quickly.”

Once land becomes dried out, there is no more evaporative cooling of the land and moistening of the atmosphere, Trenberth explains. Evaporation is the atmosphere’s natural coolant, he says, and rainfall is nature’s cleanser, pulling pollutants out of the air. Without rain and evaporation, outdoor air becomes increasingly harmful to breathe for vulnerable people.

“This [danger] is particularly important in monsoon regions in places like India, where there is a distinct wet season—four months long—and then a long dry season,” Trenberth says. Large amounts of air pollutants can build up during the long dry season, and the air pollution itself prevents the sun from penetrating through to the surface, he explains. That shuts down evaporation, and when moisture doesn’t get into the atmosphere, air pollutants don’t rain out as they do in a normal cycle.

Reduced evaporative cooling also increases the chances of heat waves, and as heat waves intensify, so do the risks of drought conditions and wildfires. Heat waves have become more frequent in some regions and are predicted to become much more common over larger geographic areas by the end of the twenty-first century.^6^ In 2003 a summer stagnation event in Western Europe resulted in intense heat, elevated O_3_ levels, and drought-related forest fires that would ultimately cause an estimated 15,000 excess deaths in France alone.^7^ Other textbook examples of this phenomenon include California’s ongoing drought, which began in 2012,^8^ and Russia’s heat wave of 2010.^9^

These enhanced dry-and-hot spells favor the creation and dispersal of airborne particulate matter, which can exacerbate asthma, rhinosinusitis, chronic obstructive pulmonary disease, and lower respiratory tract infections. All these conditions are predicted to become more widespread with climate change.^10^^,^^11^

Warmer temperatures are implicated in a different way with another air pollutant: pollen. Warmer average temperatures are already extending the length of the pollen season and the geographic range of some plant species.^12^ For example, North American ragweed, notorious for its contribution to seasonal allergies, has already made inroads in Europe and is expected to spread further as cooler areas become more hospitable.^13^ Furthermore, experimental evidence shows higher temperatures and CO_2_ levels can increase the amount and allergen content of pollen produced by individual plants.^14^

Pollen grains don’t exist long as discrete particles in the atmosphere. For instance, in times of high relative humidity, nitrogen dioxide and ground-level O_3_ can interact with birch pollen to modify some of the pollen’s proteins.^15^ *In vitro* evidence suggests these changes may produce more acute allergic responses.^16^

Ulrich Pöschl, a chemist at the Max Planck Institute for Chemistry, is now investigating how quickly increased O_3_ concentrations and humidity at ground level can modify birch-pollen proteins enough to cause a heightened allergic response. “Under clean and dry outdoor conditions, you have just a few percent of modified proteins occurring over a day,” says Poschl. “But with more O_3_ and higher humidity, the protein reactions can be quite fast, modifying dozens of percent in a day.”^17^

**From One Extreme to Another**

**Figure:**
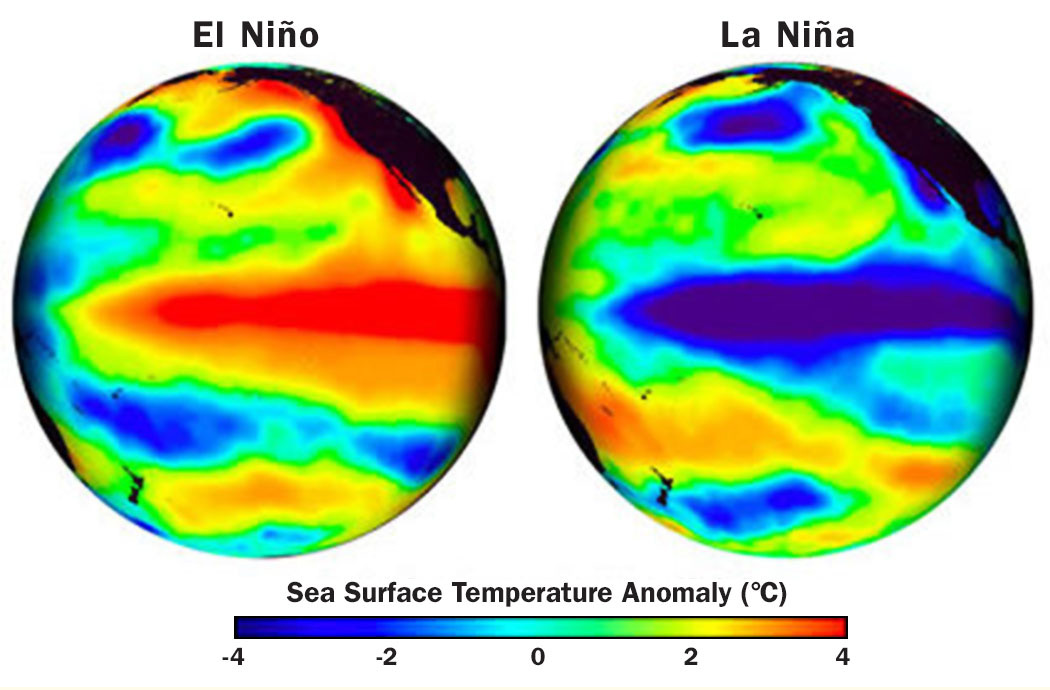

As a result of warmer surface temperatures, the atmosphere overall is becoming moister, especially above the ocean surface, because warmer air can hold more water vapor. Since the 1970s, there has been a 3.5% increase in global tropospheric water vapor associated with an average global surface temperature increase of about 0.5°C.^3^ But more moisture doesn’t necessarily mean more precipitation. Increased atmospheric water vapor can enhance climate extremes at both ends of the spectrum, contributing to more severe intense storms in some areas and more severe droughts in others.^25^

This enhanced activity can heighten the impact of the El Niño–Southern Oscillation (ENSO), the natural phenomenon with the largest impact on weather extremes around the world. ENSO occurs every three to seven years when easterly trade winds fall off and air pressure over the central equatorial Pacific Ocean becomes lower overall. The result is deviations from normal sea surface temperatures—warmer during the El Niño phase of ENSO and cooler during the El Niña phase. These fluctuations alter the normal distributions of temperatures and precipitation across the world, increasing rainfall in some regions and sharply diminishing it in others.^26^

For years scientists have studied whether climate change is altering the patterns of jet-stream winds and other global air-circulation patterns associated with ENSO. “Our estimates of changes in atmospheric circulation and the winds and so on from climate are relatively small,” says Kevin E. Trenberth, a senior scientist with the National Center for Atmospheric Research in Boulder, Colorado. “These changes are probably within the ‘noise level’ of natural variability.” In other words, for the most part weather patterns continue as they have before, with naturally occurring droughts, storms, and pressure systems.

What’s different, Trenberth says, is that the consequences of these weather patterns tend to be greater than they used to be, in part because of higher average temperatures in oceans and on land. And when an extreme in natural variability—such as an intense drought—arrives and synchronizes in the same direction as the global warming trend, the result is typically a record-breaking weather event.^26^

In 2010 and 2011 Texas suffered severe drought and outbreaks of wildfires that were likely enhanced by the appearance of La Niña, which caused the jet stream to move farther north.^27^ “We missed out on the normal complement of winter storms, and we ended up on the dry side of them,” says Texas state climatologist John Nielsen-Gammon.

The combination of a natural drought and La Niña drew moisture from the soil and helped establish a high-pressure system that parked over Texas. “When there is less soil moisture, you have higher temperatures,” says Nielsen-Gammon. This dryness, he explains, helps create high-pressure weather that enhances further drying in a positive feedback loop.

Image: Steve Albers/NASA

## Managing Air Pollution and Climate Change at the Same Time

Reducing emissions of climate pollutants to slow the pace of climate change is expected to have ancillary benefits in terms of improving air quality. In May 2015 the Climate and Clean Air Coalition (CCAC) agreed on the framework for a strategic five-year plan to maximize climate mitigation/air quality co-benefits.^1^ The CCAC is an initiative of six governments^18^ and the United Nations Environment Programme that promotes concrete, practical options for reducing short-lived climate pollutants such as methane and black carbon.^19^ The strategic framework will be formally presented at the Paris climate conference in December.

But mitigating the adverse effects of air pollution is as complex as the chemistry that produces these agents. For instance, one common air pollutant, sulfur dioxide (SO_2_), complicates efforts to simultaneously control both global warming and air quality. Power plants, vehicles, and other sources emit SO_2_ that forms sulfate particles, which harm human health but also effectively block solar radiation from reaching the surface—a cooling effect.^20^ China is seeking to reduce its SO_2_ emissions in hopes of improving urban air quality.^21^ But large reductions in SO_2_ emissions could result in enhanced global warming, Melamed says.

A sulfate particle in the air, though, would not be separate and distinct from other types of particles, and ways in which pollutants combine themselves influence how they affect climate. “You can have a particle of black carbon with a surface covering of sulfate, a pollutant that enhances cooling of the atmosphere. Or you can have a particle of sulfate with a covering of black carbon, a pollutant that increases warming,” Melamed explains. “The mixture of pollutants really matters [with respect to] how they affect climate.”

On 31 March 2015 the United States stated its intention to reduce its greenhouse gas emissions by 26–28% below 2005 levels within the next 10 years.^22^ This pledge, and those of several other nations, will be a basis for negotiations at the Paris conference, where parties aim to finally adopt a binding global agreement on measures to limit global temperature increase to below 2°C.^23^ The final negotiated agreement is proposed to come into effect in 2020.

The United States has already made steps toward leveling off its greenhouse gas emissions. Despite a 2% increase from 2012 to 2013, U.S. emissions decreased by 9% overall between 2005 and 2013.^24^ Continuing these cuts will also reduce concentrations of O_3_ and many types of PM in the atmosphere, with substantial public health benefits, says Aaron Bernstein, associate director of the Center for Health and the Global Environment at Harvard T.H. Chan School of Public Health. “If we do what we need to do to deal with climate change,” Bernstein says, “we will be healthier.”

## References

[1] CCAC. CCAC on the Road to Paris [website]. Paris, France:Clean Air Action Coalition to Reduce Short-Lived Climate Pollutants (undated). Available: http://www.unep.org/ccac/Media/PartnersInFocus/CCAContheRoadtoParis/tabid/1059945/Default.aspx [accessed 22 May 2015]

[2] Lin CYC Trends in exceedences of the ozone air quality standard in the continental United States, 1980–1998. Atmos Environ 35 19 3217 3228 2001; 10.1016/S1352-2310(01)00152-2

[3] IPCC. Technical summary. In: Climate Change 2013: The Physical Science Basis. Working Group I Contribution to the Fifth Assessment Report of the Intergovernmental Panel on Climate Change (Stocker TF, et al., eds.). Cambridge, United Kingdom and New York, NY:Cambridge University Press (2014). Available: http://www.climatechange2013.org [accessed 21 May 2015]

[4] Crutzen PJ On the background photochemistry of tropospheric ozone. Tellus A 51 1 123 146 1999; 10.1034/j.1600-0870.1999.t01-1-00010.x

[5] Sillman S The relation between ozone, NO_x_ and hydrocarbons in urban and polluted rural environments. Atmos Environ 33 12 1821 1845 1999; 10.1016/S1352-2310(98)00345-8

[6] Coumou D Robinson A Historic and future increase in the global land area affected by monthly heat extremes. Environ Res Lett 8 3 034018 2013; 10.1088/1748-9326/8/3/034018

[7] Fouillet A Excess mortality related to the August 2003 heat wave in France. Int Arch Occup Environ Health 80 1 16 24 2006; 10.1007/s00420-006-0089-4 16523319 PMC1950160

[8] Diffenbaugh MS Anthropogenic warming has increased drought risk in California. Proc Natl Acad Sci USA 112 13 3931 3936 2015; 10.1073/pnas.1422385112 25733875 PMC4386330

[9] Shaposhnikov D Mortality related to air pollution with the Moscow heat wave and wildfire of 2010. Epidemiology 25 3 359 364 2014; 10.1097/EDE.0000000000000090 24598414 PMC3984022

[10] Takaro T Climate change and respiratory health: current evidence and knowledge gaps. Expert Rev Respir Med 7 4 349 361 2013; 10.1586/17476348.2013.814367 23964626

[11] D’Amato G Climate change and respiratory diseases. Eur Respir Rev 23 132 161 169 2014; 10.1183/09059180.00001714 24881071 PMC9487563

[12] D’Amato G Allergenic pollen and pollen allergy in Europe. Allergy 62 9 976 990 2007; 10.1111/j.1398-9995.2007.01393.x 17521313

[13] Storkey J A process-based approach to predicting the effect of climate change on the distribution of an invasive allergenic plant in Europe. PLoS ONE 9 2 e88156 2014; 10.1371/journal.pone.0088156 24533071 PMC3922760

[14] Beggs PJ Adaptation to impacts of climate change on aeroallergens and allergic respiratory diseases. Int J Environ Res Public Health 7 8 3006 3021 2010; 10.3390/ijerph7083006 20948943 PMC2954564

[15] Pöschl U Shiraiwa M Multiphase chemistry at the atmosphere-biosphere interface influencing climate and public health in the Anthropocene. Chem Rev; 10.1021/cr500487s [online 9 April 2015] 25856774

[16] Gruijthuijsen YK Nitration enhances the allergenic potential of proteins. Int Arch Allergy Immunol 141 3 265 275 2006; PMID:16931888 10.1159/000095296

[17] Reinmuth-Selzle K Nitration of the birch pollen allergen Bet v 1.0101: efficiency and site-selectivity of liquid and gaseous nitrating agents. J Proteome Res 13 3 1570 1577 2014; 10.1021/pr401078h 24517313 PMC3950889

[18] Bangladesh, Canada, Ghana, Mexico, Sweden, and the United States

[19] UNEP. Governments, International Organisations, and NGOs Move to Protect Lives and the Climate from Dangerous Air Pollution [press release]. Paris, France:United Nations Environment Programme (22 May 2015). Available: http://www.unep.org/ccac/Media/PressReleases/CCACMovestoProtectLivesandtheClimate/tabid/1060207/Default.aspx [accessed 22 May 2015]

[20] Kiehl JT Briegleb BP The relative roles of sulfate aerosols and greenhouse gases in climate forcing. Science 260 5106 311 314 1993; 10.1126/science.260.5106.311 17838245

[21] Zhang Q Policy: cleaning China’s air. Nature 484 7393 161 162 2012; 10.1038/484161a 22498609

[22] UNFCCC. INDCs as Communicated by Parties [website]. Bonn, Germany:United Nations Framework Convention on Climate Change (updated 15 May 2015). Available: http://www4.unfccc.int/submissions/indc/Submission%20Pages/submissions.aspx [accessed 22 May 2015]

[23] UNFCC. Climate Change and Decisions [website]. Bonn, Germany:United Nations Framework Convention on Climate Change (2015). Available: http://www.cop21.gouv.fr/en/cop21-cmp11/climate-change-and-decisions [accessed 22 May 2015]

[24] EPA. Inventory of U.S. Greenhouse Gas Emissions and Sinks: 1990–2013. EPA 430-R-15-004. Washington, DC:U.S. Environmental Protection Agency (April 2015). Available: http://www.epa.gov/climatechange/ghgemissions/usinventoryreport.html [accessed 22 May 2015]

[25] Huntington TG Evidence for intensification of the global water cycle: review and synthesis. J Hydrol 319 1–4 83 95 2006; 10.1016/j.jhydrol.2005.07.003

[26] Trenberth KE Framing the way to relate climate extremes to climate change. Clim Change 115 2 283 290 2012; 10.1007/s10584-012-0441-5

[27] Hoerling M Anatomy of an extreme event. J Climate 26 9 2811 2832 2013; 10.1175/JCLI-D-12-00270.1

[28] Čulić V Triggering of ventricular tachycardia by meteorologic and emotional stress: protective effect of β-blockers and anxiolytics in men and elderly. Am J Epidemiol 160 11 1047 1058 2004; 10.1093/aje/kwh335 15561984

[29] Danet S Unhealthy effects of atmospheric temperature and pressure on the occurrence of myocardial infarction and coronary deaths. A 10-year survey: the Lille-World Health Organization MONICA project (Monitoring trends and determinants in cardiovascular disease). Circulation 100 1 e1 e7 1999; 10.1161/01.CIR.100.1.e1 10393689

[30] Kelly FJ Fussell JC Air pollution and airway disease. Clin Exp Allergy 41 8 1059 1071 2011; 10.1111/j.1365-2222.2011.03776.x 21623970

[31] Lim SS A comparative risk assessment of burden of disease and injury attributable to 67 risk factors and risk factor clusters in 21 regions, 1990–2010: a systematic analysis for the Global Burden of Disease Study 2010. Lancet 380 9859 2224 2260 2012; 10.1016/S0140-6736(12)61766-8 23245609 PMC4156511

[32] Jerrett M Long-term ozone exposure and mortality. N Engl J Med 360 11 1085 1095 2009; 10.1056/NEJMoa0803894 19279340 PMC4105969

[33] Ruidavets J-B Ozone air pollution is associated with acute myocardial infarction. Circulation 111 5 563 569 2005; 10.1161/01.CIR.0000154546.32135.6E 15699276

[34] Semenza JC Heat-related deaths during the July 1995 heat wave in Chicago. N Engl J Med 335 2 84 90 1996; 10.1056/NEJM199607113350203 8649494

[35] Čulić V Acute risk factors for myocardial infarction. Int J Cardiol 117 2 260 269 2007; 10.1016/j.ijcard.2006.05.011 16860887

[36] EPA. A Brief Guide to Mold, Moisture, and Your Home [website]. Washington, DC:U.S. Environmental Protection Agency (updated 3 April 2015). Availble: http://www.epa.gov/mold/moldguide.html [accessed 22 May 2015]

[37] Finlay SE Health impacts of wildfires. PLoS Curr, Edition 1 (2 November 2012); 10.1371/4f959951cce2c PMC3492003 23145351

[38] Dohrenwend PB The impact on emergency department visits for respiratory illness during the Southern California wildfires. West J Emerg Med 14 2 79 84 2013; 10.5811/westjem.2012.10.6917 23599837 PMC3628485

[39] Johnston FH Estimated global mortality attributable to smoke from landscape fires. Environ Health Perspect 120 5 695 701 2012; 10.1289/ehp.1104422 22456494 PMC3346787

[40] Hayes D Jr Bronchoconstriction triggered by breathing hot humid air in patients with asthma: role of cholinergic reflex. Am J Respir Crit Care Med 185 11 1190 1196 2012; 10.1164/rccm.201201-0088OC 22505744 PMC3373066

[41] Aitken ML Marini JJ Effect of heat delivery and extraction on airway conductance in normal and in asthmatic subjects. Am Rev Respir Dis 131 3 357 361 1985; PMID:3977173 10.1164/arrd.1985.131.3.357

[42] Mireku N Changes in weather and the effects on pediatric asthma exacerbations. Ann Allergy Asthma Immunol 103 3 220 224 2009; 10.1016/S1081-1206(10)60185-8 19788019

[43] Trenberth KE Global warming and changes in drought. Nat Clim Change 4 1 17 22 2013; 10.1038/nclimate2067

[44] Bernstein A Rice MB Lungs in a warming world: climate change and respiratory health. Chest 143 5 1455 1459 2013; 10.1378/chest.12-2384 23648909

[45] Schmier JK Ebi KL The impact of climate change and aeroallergens on children’s health. Allergy Asthma Proc 30 3 229 237 2009; 10.2500/aap.2009.30.3229 19549423

